# Author Correction: Nanoscale regulation of Ca^2+^ dependent phase transitions and real-time dynamics of SAP97/hDLG

**DOI:** 10.1038/s41467-022-32251-x

**Published:** 2022-08-01

**Authors:** Premchand Rajeev, Nivedita Singh, Adel Kechkar, Corey Butler, Narendrakumar Ramanan, Jean-Baptiste Sibarita, Mini Jose, Deepak Nair

**Affiliations:** 1 Centre for Neuroscience, Indian Institute of Science, Bangalore, Karnataka 560012 India; 2 Ecole Nationale Supérieure de Biotechnologie, Constantine, Algeria; 3 Université Bordeaux, CNRS, Interdisciplinary Institute for Neuroscience, IINS, UMR 5297, 33000 Bordeaux, France

**Keywords:** Biological physics, Nanoscale biophysics, Intrinsically disordered proteins

**Correction to**: *Nature Communications* 10.1038/s41467-022-31912-1, published online 22 July 2022

In this article the author name Corey Butler was incorrectly written as Cory Butler. The original article has been corrected.

