# Supplementary material for: Nanoscale regulation of Ca2+ dependent phase transitions and real-time dynamics of SAP97/hDLG
Source: Nat Commun. 2022 Jul 22;13:4236. doi: 10.1038/s41467-022-31912-1 (PMC9307800; doi:10.1038/s41467-022-31912-1)
Supplement: Supplementary file 1 — Supplementary Information [file 41467_2022_31912_MOESM1_ESM.docx]

**SUPPLEMENTARY FIGURES**

**Supplementary Figure 1. The effect of pharmacological treatments on the slope of bulk energy parameter bn. (A-D)** Linear regression plot of the corrected surface energy, calculated by subtracting the surface energy (an^2/3^) from the -LogP(n) data. The negative linear slope (λ) corresponds to the term b. The plot shows the effect of different treatment conditions, Thapsigargin **(B)**, w7 **(C)** and Bapta **(D)** on the slope of bn, compared to control **(A)**.

**Supplementary Figure 2. Altered nanocluster dynamics of SAP97/hDLG in Neuro-2a cells with Ca^2+^ regulation. (A-C)** The area of SAP97/hDLG clusters for control and treated conditions are plotted as a histogram. (**A**; Thapsigargin (1μM), **B**; w7 (25 μM) and **C**; Bapta (50 μM), N = 8-10 cells for control and treated datasets). (**D-I)** A total of 1000 random area of cluster data points were chosen from each dataset, rank ordered, and their linear regression is plotted for each treatment. The slope of regression depicts an arbitrary scaling factor. (**G-I**) The scaling factor provided the maximum p-value between control and scaled-treated datasets for Tg (**D,** p=0.28), w7 (**E**, p= 0.89) and Bapta (**F,** p=0.005). (**J-L).** Source data are provided as Source Data file. Cumulative frequency distributions were plotted for control, treated and scaled-treated conditions of SAP97/ hDLG in Neuro-2a cells. The datasets were analysed by KS test, **** = p < 10^-4^, *** = p<0.001, ** = p = 0.005.

**Supplementary Figure 3.**  **Relative quantification of mRNA levels of SAP97/hDLG** **in the postnatal rodent brain and in Neuro-2a cells**. (**A**) A schematic representation of the structural domains of the human homolog of SAP97/hDLG and the peptide sequence of the major splice variants. (**B-G**) Quantification of mRNA levels of N- and C-terminal splice variants of SAP97/hDLG in the postnatal (P0) mouse cortex, hippocampus (Hc) and Neuro-2a cell lines. mRNA levels corresponding to the variants, A-alone (**B**) B-alone (**C**), AB (**D**), ∆AB (**E**), I_2_ (**F**) and I_3_ (**G**) are depicted. The expression levels were normalised to β-2-microglobulin to extract ∆C_T_ and normalised again to cortical expression level to obtain ∆∆C_T_ values. The plots represent mean ± s.e.m. The data is from n= 9 cortices, 18 Hippocampi and 3 x T_75_ flask of Neuro-2a cells. Source data are provided as Source Data file

**Supplementary Figure 4. Fluorescence Recovery after photobleaching (FRAP) paradigm. (A)** Recovery of fluorescence intensity inside a region of Interest (ROI) in Neuro-2a cells expressing the C terminal splice variants of hDLG::GFP. **(B)** Normalised fluorescence recovery of I_3_-hDLG::GFP inside ROI before and after the bleaching pulse. Scale bar indicates 5µm.

**Supplementary Figure 5. The localisation of C terminal splice variants of hDLG in Neuro-2a cells using confocal microscopy.** Confocal images of Neuro-2a cells expressing hDLG variants namely, I_2_-hDLG::GFP **(A)** and I_3_-hDLG::GFP **(B)**. The images are represented as maximum intensity projections of confocal Z-stacks (∆Z=1µm). hDLG and DAPI are pseudo colour coded to green and blue in the overlay image. Scale bar indicates 5μm

**Supplementary Figure 6. Localisation and differential dynamics of C terminal splice variants of hDLG in Neuro-2a cells. (A**) Confocal images of Neuro-2a cells expressing hDLG variants namely, I_2_-hDLG::GFP (I_2_) and I_3_-hDLG::GFP (I_3_). The images are represented as maximum intensity projections of confocal stacks. hDLG and DAPI are pseudo colour coded to green and blue in the overlay image (Scale bar indicates5µm). (**B)** Recovery of fluorescence intensity after photobleaching (FRAP) paradigm on Neuro-2a cells expressing the hDLG::GFP variants. Fluorescence was normalised to the intensity of the region of interest in the pre-bleach condition. The fluorescence recovery curves were analysed using a single-phase exponential growth curve. The mobile fraction (**C)** and halftime of recovery (**D**) of hDLG constructs were extracted from the data analysis. The box represents 25% percentile, Median and 75% percentile (I_2_: 0.6569, 0.7493 and 0.8065, I_3_: 0.4875, 0.5611 and 0.8201) and the whiskers represents the minimum and maximum values (I_2_­: 0.6110 and 0.9021, I_3_­: 0.3577 and 0.9859) n=10 cells, ROI=20. The data was analysed for significance using an unpaired t-test, * = p<0.05.

**Supplementary Figure 7. Temporal variation in the fluorescence recovery of I_3_-hDLG::GFP in the presence of Calmodulin inhibitor, w7.** The experiments on live cells were performed from 1-5 min (initial) and 20 min post incubation. **(A)** The fluorescence recovery curves of I_3_ – w7 (n=9 cells), I_3_ – w7_initial_ (n=5cells) and I_3_- Tg+w7_initial_ (n=5cells). **(B, C)** One-way ANOVA with Brown Forsythe-Welsh test for multiple comparisons, for the mobile fraction and halftime of recovery of I_3_- w7, w7 _Initial_, w7 + Tg and w7 + Tg _initial_. (* = p≤ 0.05, **** = p≤ 0.0001) (Supplementary Table 7). Source data are provided as Source Data file.

**Supplementary Figure 8 Sequence alignment of the fusion proteins with BLAST.** The sequencing result of SH3-I_2_-GUK::GFP (**A**) and its sequence alignment as query with hDLG-variant 3 ( [NM_001204386.1](https://www.ncbi.nlm.nih.gov/nucleotide/NM_001204386.1?report=genbank&log$=nuclalign&blast_rank=16&RID=7M9NK6E3013)) as subject was performed using Basic Local Alignment Searching Tool (BLAST) (**B**). Sequencing result of SH3-I_3_-GUK::GFP (**C**) and its alignment as query with hDLG-variant 5 (NM_001204388.2) as subject (**D**). CaM::mCherry sequencing results (**E**) and its alignment as query with GCaMp6f (MH282426.1) as subject.

**Supplementary Figure 9 Size exclusion chromatography (SEC) for purity of fusion proteins. (A-C**) The SEC profile of SH3-I_2_-GuK::GFP (A), SH3-I_3_-GuK::GFP (B) and CaM::mCherry (C) showing the UV absorbance peak corresponding to the volume fractions of the sample (**D)** SDS-PAGE gel stained with coomassie blue for the SEC purified fractions of the fusion proteins. The SDS-PAGE gel images depicts **a.**, ladder (i), SEC purified SH3-I_2_-GuK::GFP (ii), **b.**, ladder (i), SEC purified SH3-I_3_-GuK::GFP (ii) and **c.**, ladder (i), Uninduced BL21 bacterial culture (ii), IPTG-Induced BL21 bacterial culture (iii), soluble fraction of lysate (iv) SEC purified CaM::mCherry (v). The 4 bands in CaM mCherry lane comprises of the full-length fusion protein at 47.6kDa, the full-length protein with cleaved mCherry at 37kDa, mCherry at ≈30kDa and cleaved CaM at 16kDa.

**Supplementary Figure 10** *I****n vitro* Liquid-Liquid Phase separation of SAP97 isoforms**: **A**, Top and bottom panels indicate the phase separation of C terminal isoforms, namely SH3-I_2_-GUK::GFP and SH3-I_3_-GUK::GFP, respectively in response to increasing concentration of the crowding agent, Polyethylene Glycol-4000 (PEG). The concentration of SAP97 isoforms were maintained at 1µM. Phase transitions were observed above 20% PEG in a saline buffer. Scale bar indicates 5µm.

**A.**

**Supplementary Figure 11 Absence of spontaneous phase transition for Calmodulin**: **A**, CaM::mCherry was not observed to undergo phase transition either upon increasing its concentration up to 2µM or upon increasing the concentration of PEG up to 40% in a saline buffer in the presence of 2 mM CaCl_2_. Scale bar indicates 5µm.

**Supplementary Figure 12 Concentration dependent recruitment of Calmodulin to SAP97 condensates in presence of Ca^2+^:** 1µM SH3-I_2_-GUK::GFP (**A**) or SH3-I_3_-GUK::GFP (**B**) and 2mM CaCl_2_ were co-incubated with increasing concentrations of CaM::mCherry from 100nm to 2µM. CaM::mCherry was recruited into SAP97 condensates when it was 2µM for SH3-I_2_-GUK::GFP and 1µM for SH3-I_3_-GUK::GFP. Additionally, the co-condensation of CaM with I_3_ was observed at a lower concentration of I_3_, as compared to I_2_ isoform. Scale bar indicates 5µm.

**Supplementary Figure 13 Calmodulin gets recruited to SAP97 condensates only in presence of Ca^2+^:** 1µM SH3-I_2_-GUK::GFP (**A**) or SH3-I_3_-GUK::GFP (**B**) co-incubated with CaM::mCherry (1 µm and 2 µM) in a Ca^2+^ deficient solution. CaM::mCherry failed to form condensates even though SH3-I_2_-GUK::GFP and SH3-I_3_-GUK::GFP got condensed. This shows that CaM requires both SAP97 and Ca^2+^ for recruitment into liquid-liquid phase separated domains of SAP97. Scale bar indicates 5µm.

**Supplementary Figure 14. Validating SAP97 antibody using Western blot**. Whole protein lysate from rodent brain (N=3pups) samples were run on a single PVDF membrane and cut into smaller blots for immunostaining with specific antibodies. **A.**, ladder (i), alpha Tubulin at 50kDa (1:500) (ii), **B.**, ladder (i), SAP97 at 100kDa (1:500) (ii).

**SUPPLEMENTARY TABLES**

| **Statistical Comparison** | **p value** | **Statistical Significance** |
| --- | --- | --- |
| Control vs Tg | 0.9245 | NS |
| Control vs w7 | 0.0179 | * |
| Control vs Bapta | 0.0004 | *** |

**A.**

**B.**

| **Statistical Comparison** | **p value** | **Statistical Significance** |
| --- | --- | --- |
| Control vs Tg | 0.5080 | NS |
| Control vs w7 | <0.0001 | **** |
| Control vs Bapta | <0.0001 | **** |

**Supplementary Table 1.** The table shows the p-values of multiple treatments tested for significance with respect to Control data of ∆G (**A)** and Rc **(B)** of SAP97/hDLG clusters in Neuro-2a cells.

| **Statistical Comparison** | **p value** | **Statistical Significance** |
| --- | --- | --- |
| Control vs Tg | <0.0001 | **** |
| Control vs w7 | <0.0001 | **** |
| Control vs Bapta | <0.0001 | **** |

**A.**

| **Statistical Comparison** | **p value** | **Statistical Significance** |
| --- | --- | --- |
| Control vs Tg | <0.0001 | **** |
| Control vs w7 | <0.0001 | **** |
| Control vs Bapta | <0.0001 | **** |

**B.**

**Supplementary Table 2.** The table shows the p-values of multiple treatments tested for significance with respect to Control data of average intensity (**A)** and area of clusters (**B**) of SAP97/hDLG clusters in Neuro-2a cells.

**A.**

| **Statistical Comparison** | **p value** | **Statistical Significance** |
| --- | --- | --- |
| I_2_- Control vs Tg | 0.8929 | NS |
| I_2_-Control vs w7 | <0.0001 | **** |
| I_2_-Control vs Bapta | 0.0031 | ** |

**C.**

**B.**

**D.**

| **Statistical Comparison** | **p value** | **Statistical Significance** |
| --- | --- | --- |
| I_2_- Control vs Tg | 0.0452 | * |
| I_2_-Control vs w7 | <0.0001 | **** |
| I_2_-Control vs Bapta | <0.0001 | **** |

| **Statistical Comparison** | **p value** | **Statistical Significance** |
| --- | --- | --- |
| I_3_- Control vs Tg | 0.0043 | ** |
| I_3_-Control vs w7 | 0.0155 | * |
| I_3_-Control vs Bapta | <0.0001 | **** |

| **Statistical Comparison** | **p value** | **Statistical Significance** |
| --- | --- | --- |
| I_3_- Control vs Tg | <0.0001 | **** |
| I_3_-Control vs w7 | 0.3085 | NS |
| I_3_-Control vs Bapta | <0.0001 | **** |

**Supplementary Table 3.** The table shows the p-values of multiple treatments tested for significance with respect to Control data of mobile fraction (**A)** and halftime **(B)** of I_2_- SAP97/hDLG and mobile fraction (C) and halftime (D) of I_3_- SAP97/hDLG molecules in Neuro-2a cells.

| **Statistical Comparison** | **p value** | **Statistical Significance** |
| --- | --- | --- |
| I_2_- Tg vs Tg+w7 | <0.0001 | **** |
| I_2_- Tg vs Tg+Bapta | 0.5450 | NS |

**A.**

| **Statistical Comparison** | **p value** | **Statistical Significance** |
| --- | --- | --- |
| I_2_- Tg vs Tg+w7 | 0.0005 | **** |
| I_2_- Tg vs Tg+Bapta | 0.0206 | * |

**B.**

| **Statistical Comparison** | **p value** | **Statistical Significance** |
| --- | --- | --- |
| I_3_- Tg vs Tg+w7 | 0.1768 | NS |
| I_3_- Tg vs Tg+Bapta | 0.5618 | NS |

**C.**

| **Statistical Comparison** | **p value** | **Statistical Significance** |
| --- | --- | --- |
| I_3_- Tg vs Tg+w7 | <0.0001 | **** |
| I_3_- Tg vs Tg+Bapta | <0.0001 | **** |

**D.**

**Supplementary Table 4.** The table shows the p-values of mobile fraction (**A)** and halftime **(B)** of multiple treatments tested for significance with respect to Tg treated cells over expressed with I_2_- SAP97/hDLG and mobile fraction **(C)** and halftime **(D)** of I_3_- SAP97/hDLG molecules that are ectopically expressed in Neuro-2a cells.

| **Statistical Comparison** | **p value** | **Statistical Significance** |
| --- | --- | --- |
| I_2_ vs I_3_ | <0.0001 | **** |
| I_2_-Tg vs I_3_-Tg | 0.1054 | NS |
| I_2_-w7 vs I_3_- w7 | 0.0570 | NS |
| I_2_-Bapta vs I_3_- Bapta | 0.2229 | NS |
| I_2_-Tg+w7 vs I_3_-Tg+w7 | 0.0213 | * |
| I_2_-Tg+Bapta vs I_3_-Tg+Bapta | 0.0006 | *** |

**A.**

**B.**

| **Statistical Comparison** | **p value** | **Statistical Significance** |
| --- | --- | --- |
| I_2_ vs I_3_ | 0.2769 | NS |
| I_2_-Tg vs I_3_-Tg | <0.0001 | **** |
| I_2_-w7 vs I_3_- w7 | 0.0023 | ** |
| I_2_-Bapta vs I_3_- Bapta | 0.0069 | ** |
| I_2_-Tg+w7 vs I_3_-Tg+w7 | 0.0011 | ** |
| I_2_-Tg+Bapta vs I_3_-Tg+Bapta | <0.0001 | **** |

**Supplementary Table 5.** The table shows the p-values of multiple statistical comparisons of mobile fraction (**A)** and halftime **(B)** of multiple treatments on ectopically expressed SAP97/hDLG molecules in Neuro-2a cells.

| **Statistical Comparison** | **p value** | **Statistical Significance** |
| --- | --- | --- |
| Control vs. I_2_ | 0.0040 | **** |
| Control vs. I_3_ | 0.1209 | * |
| Control vs. 14-DIV | 0.0009 | * |
| I_2_ vs. I_3_ | 0.3945 | * |
| I_2_ vs. 14-DIV | <0.0001 | * |
| I_3_ vs. 14-DIV | <0.0001 | NS |

**A.**

| **Statistical Comparison** | **p value** | **Statistical Significance** |
| --- | --- | --- |
| Control vs. I_2_ | <0.0001 | ** |
| Control vs. I_3_ | 0.0250 | NS |
| Control vs. 14-DIV | 0.0144 | NS |
| I_2_ vs. I_3_ | 0.0300 | *** |
| I_2_ vs. 14-DIV | 0.0258 | **** |
| I_3_ vs. 14-DIV | >0.9999 | **** |

**B.**

**Supplementary Table 6.** The table shows the p-values of multiple treatments tested for significance with respect to Control data of Rc (**A)** and ∆G **(B)** of SAP97/hDLG clusters in 14-DIV old hippocampal pyramidal neurons.

**A.**

| **Statistical Comparison** | **p value** | **Statistical Significance** |
| --- | --- | --- |
| I3 vs. w7 | 0.7990 | NS |
| I3 vs. w7 _initial_ | 0.2680 | NS |
| Tg vs. Tg+w7 | 0.3000 | NS |
| Tg vs. Tg+w7 _initial_ | 0.8060 | NS |

| **Statistical Comparison** | **p value** | **Statistical Significance** |
| --- | --- | --- |
| I3 vs. w7 | 0.6266 | NS |
| I3 vs. w7 _initial_ | 0.0783 | NS |
| Tg vs. Tg+w7 | <0.0001 | **** |
| Tg vs. Tg+w7 _initial_ | 0.0067 | ** |

**B.**

**Supplementary Table 7.** The table shows the p-values of multiple statistical comparisons of mobile fraction (**A)** and halftime **(B)** of multiple treatments on ectopically expressed SAP97/hDLG molecules in Neuro-2a cells.

| **Sl no.** | **Primers** | **Sequence (5’-3’)** | **Tm (⁰C)** | **Usage** |
| --- | --- | --- | --- | --- |
| 1 | SAP97/hDLG -A-FWD | ACCAATAAAGCCCACAGAAGC | 55.7 | qRT-PCR |
| 2 | SAP97/hDLG -A-REV | CATCAGTGCCATTAACCTGTG | 54.5 | qRT-PCR |
| 3 | SAP97/hDLG -B-FWD | ACCAATAAAGGCAAATCCTCCTCC | 56.8 | qRT-PCR |
| 4 | SAP97/hDLG -B-REV | ACCAAGACCCGAATTTCCCC | 57.5 | qRT-PCR |
| 5 | SAP97/hDLG -AB-FWD | ACCAATAAAGCCCACAGAAGC | 55.7 | qRT-PCR |
| 6 | SAP97/hDLG -AB-REV | AAGTTGGTGTCTCTAAGCTG | 52.4 | qRT-PCR |
| 7 | SAP97/hDLG -∆AB-FWD | CACCAATAAAGGTTAATGGCAC | 52.3 | qRT-PCR |
| 8 | SAP97/hDLG -∆AB -REV | CCGCCTGTGATAATTTTGG | 51.6 | qRT-PCR |
| 9 | SAP97/hDLG -I_2_- FWD | ACAAGAGGAGATAAAGGGGAGA | 54.7 | qRT-PCR |
| 10 | SAP97/hDLG -I_2_- REV | TGACCACGGTAACTACTTTCACT | 55.6 | qRT-PCR |
| 11 | SAP97/hDLG -I_3_-FWD | AGAGGAGATAAAGGGCAGTC | 53.5 | qRT-PCR |
| 12 | SAP97/hDLG -I_3_-REV | CATTAGAAGTTACGTGCTGGTCA | 54.8 | qRT-PCR |
| 13 | SAP97/hDLG::GFP-FWD | CAACCACTACCTGAGCAC | 53 | Sequencing hDLG |
| 14 | SAP97/hDLG::GFP-REV | AAACCACAACTAGAATGCAG | 51 | Sequencing hDLG |
| 15 | GFP-Not1-FWD | ATCGCGGCCGCATGGTGAGCAAGGGC | 65 | Subcloning SAP97/hDLG fusion construct |
| 16 | GFP-Xho1-REV | ATACTCGAGTCACTTGTACAGCTCGTCCATGCC | 67 | Subcloning SAP97/hDLG fusion construct |
| 17 | SAP97/hDLG-SalI-FWD | ATAGTCGACATCGATCCCTCTATGTCAGAGCCC | 65 | Subcloning SAP97/hDLG fusion construct |
| 18 | SAP97/hDLG-HindIII-REV | ATCAAGCTTGTAGCTTTTCTTTTGCCGGAACCC | 65 | Subcloning SAP97/hDLG fusion construct |
| 19 | Pet28a- SAP97/hDLG -Seq-FWD | GGAATTGTGAGCGGATAACAATTCCC | 59 | Sequencing SAP97/hDLG fusion construct |
| 20 | Pet28a- SAP97/hDLG -Seq-REV | TCGTACAGACTGAACACTTGTTCC | 58 | Sequencing SAP97/hDLG fusion construct |

**Supplementary Table 8.** The table shows the primers/oligonucleotides used in multiple experiments in the study.

| **Sl no.** | **Construct** | **Sequence (5’-3’)** |
| --- | --- | --- |
| 1 | pET-28a-SH3-I_2_-GUK::GFP | TGGCGAATGGGACGCGCCCTGTAGCGGCGCATTAAGCGCGGCGGGTGTGGTGGTTACGCGCAGCGTGACCGCTACACTTGCCAGCGCCCTAGCGCCCGCTCCTTTCGCTTTCTTCCCTTCCTTTCTCGCCACGTTCGCCGGCTTTCCCCGTCAAGCTCTAAATCGGGGGCTCCCTTTAGGGTTCCGATTTAGTGCTTTACGGCACCTCGACCCCAAAAAACTTGATTAGGGTGATGGTTCACGTAGTGGGCCATCGCCCTGATAGACGGTTTTTCGCCCTTTGACGTTGGAGTCCACGTTCTTTAATAGTGGACTCTTGTTCCAAACTGGAACAACACTCAACCCTATCTCGGTCTATTCTTTTGATTTATAAGGGATTTTGCCGATTTCGGCCTATTGGTTAAAAAATGAGCTGATTTAACAAAAATTTAACGCGAATTTTAACAAAATATTAACGTTTACAATTTCAGGTGGCACTTTTCGGGGAAATGTGCGCGGAACCCCTATTTGTTTATTTTTCTAAATACATTCAAATATGTATCCGCTCATGAATTAATTCTTAGAAAAACTCATCGAGCATCAAATGAAACTGCAATTTATTCATATCAGGATTATCAATACCATATTTTTGAAAAAGCCGTTTCTGTAATGAAGGAGAAAACTCACCGAGGCAGTTCCATAGGATGGCAAGATCCTGGTATCGGTCTGCGATTCCGACTCGTCCAACATCAATACAACCTATTAATTTCCCCTCGTCAAAAATAAGGTTATCAAGTGAGAAATCACCATGAGTGACGACTGAATCCGGTGAGAATGGCAAAAGTTTATGCATTTCTTTCCAGACTTGTTCAACAGGCCAGCCATTACGCTCGTCATCAAAATCACTCGCATCAACCAAACCGTTATTCATTCGTGATTGCGCCTGAGCGAGACGAAATACGCGATCGCTGTTAAAAGGACAATTACAAACAGGAATCGAATGCAACCGGCGCAGGAACACTGCCAGCGCATCAACAATATTTTCACCTGAATCAGGATATTCTTCTAATACCTGGAATGCTGTTTTCCCGGGGATCGCAGTGGTGAGTAACCATGCATCATCAGGAGTACGGATAAAATGCTTGATGGTCGGAAGAGGCATAAATTCCGTCAGCCAGTTTAGTCTGACCATCTCATCTGTAACATCATTGGCAACGCTACCTTTGCCATGTTTCAGAAACAACTCTGGCGCATCGGGCTTCCCATACAATCGATAGATTGTCGCACCTGATTGCCCGACATTATCGCGAGCCCATTTATACCCATATAAATCAGCATCCATGTTGGAATTTAATCGCGGCCTAGAGCAAGACGTTTCCCGTTGAATATGGCTCATAACACCCCTTGTATTACTGTTTATGTAAGCAGACAGTTTTATTGTTCATGACCAAAATCCCTTAACGTGAGTTTTCGTTCCACTGAGCGTCAGACCCCGTAGAAAAGATCAAAGGATCTTCTTGAGATCCTTTTTTTCTGCGCGTAATCTGCTGCTTGCAAACAAAAAAACCACCGCTACCAGCGGTGGTTTGTTTGCCGGATCAAGAGCTACCAACTCTTTTTCCGAAGGTAACTGGCTTCAGCAGAGCGCAGATACCAAATACTGTCCTTCTAGTGTAGCCGTAGTTAGGCCACCACTTCAAGAACTCTGTAGCACCGCCTACATACCTCGCTCTGCTAATCCTGTTACCAGTGGCTGCTGCCAGTGGCGATAAGTCGTGTCTTACCGGGTTGGACTCAAGACGATAGTTACCGGATAAGGCGCAGCGGTCGGGCTGAACGGGGGGTTCGTGCACACAGCCCAGCTTGGAGCGAACGACCTACACCGAACTGAGATACCTACAGCGTGAGCTATGAGAAAGCGCCACGCTTCCCGAAGGGAGAAAGGCGGACAGGTATCCGGTAAGCGGCAGGGTCGGAACAGGAGAGCGCACGAGGGAGCTTCCAGGGGGAAACGCCTGGTATCTTTATAGTCCTGTCGGGTTTCGCCACCTCTGACTTGAGCGTCGATTTTTGTGATGCTCGTCAGGGGGGCGGAGCCTATGGAAAAACGCCAGCAACGCGGCCTTTTTACGGTTCCTGGCCTTTTGCTGGCCTTTTGCTCACATGTTCTTTCCTGCGTTATCCCCTGATTCTGTGGATAACCGTATTACCGCCTTTGAGTGAGCTGATACCGCTCGCCGCAGCCGAACGACCGAGCGCAGCGAGTCAGTGAGCGAGGAAGCGGAAGAGCGCCTGATGCGGTATTTTCTCCTTACGCATCTGTGCGGTATTTCACACCGCATATATGGTGCACTCTCAGTACAATCTGCTCTGATGCCGCATAGTTAAGCCAGTATACACTCCGCTATCGCTACGTGACTGGGTCATGGCTGCGCCCCGACACCCGCCAACACCCGCTGACGCGCCCTGACGGGCTTGTCTGCTCCCGGCATCCGCTTACAGACAAGCTGTGACCGTCTCCGGGAGCTGCATGTGTCAGAGGTTTTCACCGTCATCACCGAAACGCGCGAGGCAGCTGCGGTAAAGCTCATCAGCGTGGTCGTGAAGCGATTCACAGATGTCTGCCTGTTCATCCGCGTCCAGCTCGTTGAGTTTCTCCAGAAGCGTTAATGTCTGGCTTCTGATAAAGCGGGCCATGTTAAGGGCGGTTTTTTCCTGTTTGGTCACTGATGCCTCCGTGTAAGGGGGATTTCTGTTCATGGGGGTAATGATACCGATGAAACGAGAGAGGATGCTCACGATACGGGTTACTGATGATGAACATGCCCGGTTACTGGAACGTTGTGAGGGTAAACAACTGGCGGTATGGATGCGGCGGGACCAGAGAAAAATCACTCAGGGTCAATGCCAGCGCTTCGTTAATACAGATGTAGGTGTTCCACAGGGTAGCCAGCAGCATCCTGCGATGCAGATCCGGAACATAATGGTGCAGGGCGCTGACTTCCGCGTTTCCAGACTTTACGAAACACGGAAACCGAAGACCATTCATGTTGTTGCTCAGGTCGCAGACGTTTTGCAGCAGCAGTCGCTTCACGTTCGCTCGCGTATCGGTGATTCATTCTGCTAACCAGTAAGGCAACCCCGCCAGCCTAGCCGGGTCCTCAACGACAGGAGCACGATCATGCGCACCCGTGGGGCCGCCATGCCGGCGATAATGGCCTGCTTCTCGCCGAAACGTTTGGTGGCGGGACCAGTGACGAAGGCTTGAGCGAGGGCGTGCAAGATTCCGAATACCGCAAGCGACAGGCCGATCATCGTCGCGCTCCAGCGAAAGCGGTCCTCGCCGAAAATGACCCAGAGCGCTGCCGGCACCTGTCCTACGAGTTGCATGATAAAGAAGACAGTCATAAGTGCGGCGACGATAGTCATGCCCCGCGCCCACCGGAAGGAGCTGACTGGGTTGAAGGCTCTCAAGGGCATCGGTCGAGATCCCGGTGCCTAATGAGTGAGCTAACTTACATTAATTGCGTTGCGCTCACTGCCCGCTTTCCAGTCGGGAAACCTGTCGTGCCAGCTGCATTAATGAATCGGCCAACGCGCGGGGAGAGGCGGTTTGCGTATTGGGCGCCAGGGTGGTTTTTCTTTTCACCAGTGAGACGGGCAACAGCTGATTGCCCTTCACCGCCTGGCCCTGAGAGAGTTGCAGCAAGCGGTCCACGCTGGTTTGCCCCAGCAGGCGAAAATCCTGTTTGATGGTGGTTAACGGCGGGATATAACATGAGCTGTCTTCGGTATCGTCGTATCCCACTACCGAGATATCCGCACCAACGCGCAGCCCGGACTCGGTAATGGCGCGCATTGCGCCCAGCGCCATCTGATCGTTGGCAACCAGCATCGCAGTGGGAACGATGCCCTCATTCAGCATTTGCATGGTTTGTTGAAAACCGGACATGGCACTCCAGTCGCCTTCCCGTTCCGCTATCGGCTGAATTTGATTGCGAGTGAGATATTTATGCCAGCCAGCCAGACGCAGACGCGCCGAGACAGAACTTAATGGGCCCGCTAACAGCGCGATTTGCTGGTGACCCAATGCGACCAGATGCTCCACGCCCAGTCGCGTACCGTCTTCATGGGAGAAAATAATACTGTTGATGGGTGTCTGGTCAGAGACATCAAGAAATAACGCCGGAACATTAGTGCAGGCAGCTTCCACAGCAATGGCATCCTGGTCATCCAGCGGATAGTTAATGATCAGCCCACTGACGCGTTGCGCGAGAAGATTGTGCACCGCCGCTTTACAGGCTTCGACGCCGCTTCGTTCTACCATCGACACCACCACGCTGGCACCCAGTTGATCGGCGCGAGATTTAATCGCCGCGACAATTTGCGACGGCGCGTGCAGGGCCAGACTGGAGGTGGCAACGCCAATCAGCAACGACTGTTTGCCCGCCAGTTGTTGTGCCACGCGGTTGGGAATGTAATTCAGCTCCGCCATCGCCGCTTCCACTTTTTCCCGCGTTTTCGCAGAAACGTGGCTGGCCTGGTTCACCACGCGGGAAACGGTCTGATAAGAGACACCGGCATACTCTGCGACATCGTATAACGTTACTGGTTTCACATTCACCACCCTGAATTGACTCTCTTCCGGGCGCTATCATGCCATACCGCGAAAGGTTTTGCGCCATTCGATGGTGTCCGGGATCTCGACGCTCTCCCTTATGCGACTCCTGCATTAGGAAGCAGCCCAGTAGTAGGTTGAGGCCGTTGAGCACCGCCGCCGCAAGGAATGGTGCATGCAAGGAGATGGCGCCCAACAGTCCCCCGGCCACGGGGCCTGCCACCATACCCACGCCGAAACAAGCGCTCATGAGCCCGAAGTGGCGAGCCCGATCTTCCCCATCGGTGATGTCGGCGATATAGGCGCCAGCAACCGCACCTGTGGCGCCGGTGATGCCGGCCACGATGCGTCCGGCGTAGAGGATCGAGATCTCGATCCCGCGAAATTAATACGACTCACTATAGGGGAATTGTGAGCGGATAACAATTCCCCTCTAGAAATAATTTTGTTTAACTTTAAGAAGGAGATATACCATGGGCAGCAGCCATCATCATCATCATCACAGCAGCGGCCTGGTGCCGCGCGGCAGCCATATGGCTAGCATGACTGGTGGACAGCAAATGGGTCGCGGATCCGAATTCGAGCTCCGTCGACATCGATCCCTCTATGTCAGAGCCCTTTTcGATTATGACAAGACTAAAGACAGTGGGCTTCCCAGTCAGGGACTGAACTTCAAATTTGGAGATATCCTCCATGTTATTAATGCTTCTGATGATGAATGGTGGCAAGCCAGGCAGGTTACACCAGATGGTGAGAGCGATGAGGTCGGAGTGATTCCCAGTAAACGCAGAGTTGAGAAGAAAGAACGAGCCCGATTAAAAACAGTGAAATTCAATTCTAAAACGAGAGATAAAGGGGAGATCCCTGACGACATGGGATCAAAAGGCCTGAAGCATGTAACTTCTAATGCCAGCGATAGTGAAAGTAGTTACCGTGGTCAAGAAGAATACGTCTTATCTTATGAACCAGTGAATCAACAAGAAGTTAATTATACTCGACCAGTGATCATATTGGGACCTATGAAAGACAGGATAAATGATGACTTGATCTCAGAATTTCCTGACAAATTTGGATCCTGTGTTCCTCATACAACTAGACCAAAACGAGATTATGAGGTAGATGGAAGAGATTATCATTTTGTGACTTCAAGAGAGCAGATGGAAAAAGATATCCAGGAACATAAATTCATTGAAGCTGGCCAGTATAACAATCATCTATATGGAACAAGTGTTCAGTCTGTACGAGAAGTAGCAGAAAAGGGCAAACACTGTATCCTTGATGTGTCTGGAAATGCCATAAAGAGATTACAGATTGCACAGCTTTACCCTATCTCCATTTTTATTAAACCCAAATCCATGGAAAATATCATGGAAATGAATAAGCGTCTAACAGAAGAACAAGCCAGAAAAACATTTGAGAGAGCCATGAAACTGGAACAGGAGTTTACTGAACATTTCACAGCTATTGTACAGGGGGATACGCTGGAAGACATTTACAACCAAGTGAAACAGATCATAGAAGAACAATCTGGTTCTTACATCTGGGTTCCGGCAAAAGAAAAGCTACAAGCTTGCGGCCGCATGGTGAGCAAGGGCGAGGAGCTGTTCACCGGGGTGGTGCCCATCCTGGTCGAGCTGGACGGCGACGTAAACGGCCACAAGTTCAGCGTGTCCGGCGAGGGCGAGGGCGATGCCACCTACGGCAAGCTGACCCTGAAGTTCATCTGCACCACCGGCAAGCTGCCCGTGCCCTGGCCCACCCTCGTGACCACCCTGACCTACGGCGTGCAGTGCTTCAGCCGCTACCCCGACCACATGAAGCAGCACGACTTCTTCAAGTCCGCCATGCCCGAAGGCTACGTCCAGGAGCGCACCATCTTCTTCAAGGACGACGGCAACTACAAGACCCGCGCCGAGGTGAAGTTCGAGGGCGACACCCTGGTGAACCGCATCGAGCTGAAGGGCATCGACTTCAAGGAGGACGGCAACATCCTGGGGCACAAGCTGGAGTACAACTACAACAGCCACAACGTCTATATCATGGCCGACAAGCAGAAGAACGGCATCAAGGTGAACTTCAAGATCCGCCACAACATCGAGGACGGCAGCGTGCAGCTCGCCGACCACTACCAGCAGAACACCCCCATCGGCGACGGCCCCGTGCTGCTGCCCGACAACCACTACCTGAGCACCCAGTCCGCCCTGAGCAAAGACCCCAACGAGAAGCGCGATCACATGGTCCTGCTGGAGTTCGTGACCGCCGCCGGGATCACTCTCGGCATGGACGAGCTGTACAAGTGACTCGAGCACCACCACCACCACCACTGAGATCCGGCTGCTAACAAAGCCCGAAAGGAAGCTGAGTTGGCTGCTGCCACCGCTGAGCAATAACTAGCATAACCCCTTGGGGCCTCTAAACGGGTCTTGAGGGGTTTTTTGCTGAAAGGAGGAACTATATCCGGAT |
| 2 | pET-28a-SH3-I_3_-GUK::GFP | TGGCGAATGGGACGCGCCCTGTAGCGGCGCATTAAGCGCGGCGGGTGTGGTGGTTACGCGCAGCGTGACCGCTACACTTGCCAGCGCCCTAGCGCCCGCTCCTTTCGCTTTCTTCCCTTCCTTTCTCGCCACGTTCGCCGGCTTTCCCCGTCAAGCTCTAAATCGGGGGCTCCCTTTAGGGTTCCGATTTAGTGCTTTACGGCACCTCGACCCCAAAAAACTTGATTAGGGTGATGGTTCACGTAGTGGGCCATCGCCCTGATAGACGGTTTTTCGCCCTTTGACGTTGGAGTCCACGTTCTTTAATAGTGGACTCTTGTTCCAAACTGGAACAACACTCAACCCTATCTCGGTCTATTCTTTTGATTTATAAGGGATTTTGCCGATTTCGGCCTATTGGTTAAAAAATGAGCTGATTTAACAAAAATTTAACGCGAATTTTAACAAAATATTAACGTTTACAATTTCAGGTGGCACTTTTCGGGGAAATGTGCGCGGAACCCCTATTTGTTTATTTTTCTAAATACATTCAAATATGTATCCGCTCATGAATTAATTCTTAGAAAAACTCATCGAGCATCAAATGAAACTGCAATTTATTCATATCAGGATTATCAATACCATATTTTTGAAAAAGCCGTTTCTGTAATGAAGGAGAAAACTCACCGAGGCAGTTCCATAGGATGGCAAGATCCTGGTATCGGTCTGCGATTCCGACTCGTCCAACATCAATACAACCTATTAATTTCCCCTCGTCAAAAATAAGGTTATCAAGTGAGAAATCACCATGAGTGACGACTGAATCCGGTGAGAATGGCAAAAGTTTATGCATTTCTTTCCAGACTTGTTCAACAGGCCAGCCATTACGCTCGTCATCAAAATCACTCGCATCAACCAAACCGTTATTCATTCGTGATTGCGCCTGAGCGAGACGAAATACGCGATCGCTGTTAAAAGGACAATTACAAACAGGAATCGAATGCAACCGGCGCAGGAACACTGCCAGCGCATCAACAATATTTTCACCTGAATCAGGATATTCTTCTAATACCTGGAATGCTGTTTTCCCGGGGATCGCAGTGGTGAGTAACCATGCATCATCAGGAGTACGGATAAAATGCTTGATGGTCGGAAGAGGCATAAATTCCGTCAGCCAGTTTAGTCTGACCATCTCATCTGTAACATCATTGGCAACGCTACCTTTGCCATGTTTCAGAAACAACTCTGGCGCATCGGGCTTCCCATACAATCGATAGATTGTCGCACCTGATTGCCCGACATTATCGCGAGCCCATTTATACCCATATAAATCAGCATCCATGTTGGAATTTAATCGCGGCCTAGAGCAAGACGTTTCCCGTTGAATATGGCTCATAACACCCCTTGTATTACTGTTTATGTAAGCAGACAGTTTTATTGTTCATGACCAAAATCCCTTAACGTGAGTTTTCGTTCCACTGAGCGTCAGACCCCGTAGAAAAGATCAAAGGATCTTCTTGAGATCCTTTTTTTCTGCGCGTAATCTGCTGCTTGCAAACAAAAAAACCACCGCTACCAGCGGTGGTTTGTTTGCCGGATCAAGAGCTACCAACTCTTTTTCCGAAGGTAACTGGCTTCAGCAGAGCGCAGATACCAAATACTGTCCTTCTAGTGTAGCCGTAGTTAGGCCACCACTTCAAGAACTCTGTAGCACCGCCTACATACCTCGCTCTGCTAATCCTGTTACCAGTGGCTGCTGCCAGTGGCGATAAGTCGTGTCTTACCGGGTTGGACTCAAGACGATAGTTACCGGATAAGGCGCAGCGGTCGGGCTGAACGGGGGGTTCGTGCACACAGCCCAGCTTGGAGCGAACGACCTACACCGAACTGAGATACCTACAGCGTGAGCTATGAGAAAGCGCCACGCTTCCCGAAGGGAGAAAGGCGGACAGGTATCCGGTAAGCGGCAGGGTCGGAACAGGAGAGCGCACGAGGGAGCTTCCAGGGGGAAACGCCTGGTATCTTTATAGTCCTGTCGGGTTTCGCCACCTCTGACTTGAGCGTCGATTTTTGTGATGCTCGTCAGGGGGGCGGAGCCTATGGAAAAACGCCAGCAACGCGGCCTTTTTACGGTTCCTGGCCTTTTGCTGGCCTTTTGCTCACATGTTCTTTCCTGCGTTATCCCCTGATTCTGTGGATAACCGTATTACCGCCTTTGAGTGAGCTGATACCGCTCGCCGCAGCCGAACGACCGAGCGCAGCGAGTCAGTGAGCGAGGAAGCGGAAGAGCGCCTGATGCGGTATTTTCTCCTTACGCATCTGTGCGGTATTTCACACCGCATATATGGTGCACTCTCAGTACAATCTGCTCTGATGCCGCATAGTTAAGCCAGTATACACTCCGCTATCGCTACGTGACTGGGTCATGGCTGCGCCCCGACACCCGCCAACACCCGCTGACGCGCCCTGACGGGCTTGTCTGCTCCCGGCATCCGCTTACAGACAAGCTGTGACCGTCTCCGGGAGCTGCATGTGTCAGAGGTTTTCACCGTCATCACCGAAACGCGCGAGGCAGCTGCGGTAAAGCTCATCAGCGTGGTCGTGAAGCGATTCACAGATGTCTGCCTGTTCATCCGCGTCCAGCTCGTTGAGTTTCTCCAGAAGCGTTAATGTCTGGCTTCTGATAAAGCGGGCCATGTTAAGGGCGGTTTTTTCCTGTTTGGTCACTGATGCCTCCGTGTAAGGGGGATTTCTGTTCATGGGGGTAATGATACCGATGAAACGAGAGAGGATGCTCACGATACGGGTTACTGATGATGAACATGCCCGGTTACTGGAACGTTGTGAGGGTAAACAACTGGCGGTATGGATGCGGCGGGACCAGAGAAAAATCACTCAGGGTCAATGCCAGCGCTTCGTTAATACAGATGTAGGTGTTCCACAGGGTAGCCAGCAGCATCCTGCGATGCAGATCCGGAACATAATGGTGCAGGGCGCTGACTTCCGCGTTTCCAGACTTTACGAAACACGGAAACCGAAGACCATTCATGTTGTTGCTCAGGTCGCAGACGTTTTGCAGCAGCAGTCGCTTCACGTTCGCTCGCGTATCGGTGATTCATTCTGCTAACCAGTAAGGCAACCCCGCCAGCCTAGCCGGGTCCTCAACGACAGGAGCACGATCATGCGCACCCGTGGGGCCGCCATGCCGGCGATAATGGCCTGCTTCTCGCCGAAACGTTTGGTGGCGGGACCAGTGACGAAGGCTTGAGCGAGGGCGTGCAAGATTCCGAATACCGCAAGCGACAGGCCGATCATCGTCGCGCTCCAGCGAAAGCGGTCCTCGCCGAAAATGACCCAGAGCGCTGCCGGCACCTGTCCTACGAGTTGCATGATAAAGAAGACAGTCATAAGTGCGGCGACGATAGTCATGCCCCGCGCCCACCGGAAGGAGCTGACTGGGTTGAAGGCTCTCAAGGGCATCGGTCGAGATCCCGGTGCCTAATGAGTGAGCTAACTTACATTAATTGCGTTGCGCTCACTGCCCGCTTTCCAGTCGGGAAACCTGTCGTGCCAGCTGCATTAATGAATCGGCCAACGCGCGGGGAGAGGCGGTTTGCGTATTGGGCGCCAGGGTGGTTTTTCTTTTCACCAGTGAGACGGGCAACAGCTGATTGCCCTTCACCGCCTGGCCCTGAGAGAGTTGCAGCAAGCGGTCCACGCTGGTTTGCCCCAGCAGGCGAAAATCCTGTTTGATGGTGGTTAACGGCGGGATATAACATGAGCTGTCTTCGGTATCGTCGTATCCCACTACCGAGATATCCGCACCAACGCGCAGCCCGGACTCGGTAATGGCGCGCATTGCGCCCAGCGCCATCTGATCGTTGGCAACCAGCATCGCAGTGGGAACGATGCCCTCATTCAGCATTTGCATGGTTTGTTGAAAACCGGACATGGCACTCCAGTCGCCTTCCCGTTCCGCTATCGGCTGAATTTGATTGCGAGTGAGATATTTATGCCAGCCAGCCAGACGCAGACGCGCCGAGACAGAACTTAATGGGCCCGCTAACAGCGCGATTTGCTGGTGACCCAATGCGACCAGATGCTCCACGCCCAGTCGCGTACCGTCTTCATGGGAGAAAATAATACTGTTGATGGGTGTCTGGTCAGAGACATCAAGAAATAACGCCGGAACATTAGTGCAGGCAGCTTCCACAGCAATGGCATCCTGGTCATCCAGCGGATAGTTAATGATCAGCCCACTGACGCGTTGCGCGAGAAGATTGTGCACCGCCGCTTTACAGGCTTCGACGCCGCTTCGTTCTACCATCGACACCACCACGCTGGCACCCAGTTGATCGGCGCGAGATTTAATCGCCGCGACAATTTGCGACGGCGCGTGCAGGGCCAGACTGGAGGTGGCAACGCCAATCAGCAACGACTGTTTGCCCGCCAGTTGTTGTGCCACGCGGTTGGGAATGTAATTCAGCTCCGCCATCGCCGCTTCCACTTTTTCCCGCGTTTTCGCAGAAACGTGGCTGGCCTGGTTCACCACGCGGGAAACGGTCTGATAAGAGACACCGGCATACTCTGCGACATCGTATAACGTTACTGGTTTCACATTCACCACCCTGAATTGACTCTCTTCCGGGCGCTATCATGCCATACCGCGAAAGGTTTTGCGCCATTCGATGGTGTCCGGGATCTCGACGCTCTCCCTTATGCGACTCCTGCATTAGGAAGCAGCCCAGTAGTAGGTTGAGGCCGTTGAGCACCGCCGCCGCAAGGAATGGTGCATGCAAGGAGATGGCGCCCAACAGTCCCCCGGCCACGGGGCCTGCCACCATACCCACGCCGAAACAAGCGCTCATGAGCCCGAAGTGGCGAGCCCGATCTTCCCCATCGGTGATGTCGGCGATATAGGCGCCAGCAACCGCACCTGTGGCGCCGGTGATGCCGGCCACGATGCGTCCGGCGTAGAGGATCGAGATCTCGATCCCGCGAAATTAATACGACTCACTATAGGGGAATTGTGAGCGGATAACAATTCCCCTCTAGAAATAATTTTGTTTAACTTTAAGAAGGAGATATACCATGGGCAGCAGCCATCATCATCATCATCACAGCAGCGGCCTGGTGCCGCGCGGCAGCCATATGGCTAGCATGACTGGTGGACAGCAAATGGGTCGCGGATCCGAATTCGAGCTCCGTCGACATCGATCCCTCTATGTCAGAGCCCTTTTcGATTATGACAAGACTAAAGACAGTGGGCTTCCCAGTCAGGGACTGAACTTCAAATTTGGAGATATCCTCCATGTTATTAATGCTTCTGATGATGAATGGTGGCAAGCCAGGCAGGTTACACCAGATGGTGAGAGCGATGAGGTCGGAGTGATTCCCAGTAAACGCAGAGTTGAGAAGAAAGAACGAGCCCGATTAAAAACAGTGAAATTCAATTCTAAAACGAGAGATAAAGGGCAGTCATTCAATGACAAGCGTAAAAAGAACCTCTTTTCCCGAAAATTTCCCTTCTACAAGAACAAGGACCAGAGTGAACAGGAAACGAGTGATGCTGACGAGATCCCTGACGACATGGGATCAAAAGGCCTGAAGCATGTAACTTCTAATGCCAGCGATAGTGAAAGTAGTTACCGTGGTCAAGAAGAATACGTCTTATCTTATGAACCAGTGAATCAACAAGAAGTTAATTATACTCGACCAGTGATCATATTGGGACCTATGAAAGACAGGATAAATGATGACTTGATCTCAGAATTTCCTGACAAATTTGGATCCTGTGTTCCTCATACAACTAGACCAAAACGAGATTATGAGGTAGATGGAAGAGATTATCATTTTGTGACTTCAAGAGAGCAGATGGAAAAAGATATCCAGGAACATAAATTCATTGAAGCTGGCCAGTATAACAATCATCTATATGGAACAAGTGTTCAGTCTGTACGAGAAGTAGCAGAAAAGGGCAAACACTGTATCCTTGATGTGTCTGGAAATGCCATAAAGAGATTACAGATTGCACAGCTTTACCCTATCTCCATTTTTATTAAACCCAAATCCATGGAAAATATCATGGAAATGAATAAGCGTCTAACAGAAGAACAAGCCAGAAAAACATTTGAGAGAGCCATGAAACTGGAACAGGAGTTTACTGAACATTTCACAGCTATTGTACAGGGGGATACGCTGGAAGACATTTACAACCAAGTGAAACAGATCATAGAAGAACAATCTGGTTCTTACATCTGGGTTCCGGCAAAAGAAAAGCTACAAGCTTGCGGCCGCATGGTGAGCAAGGGCGAGGAGCTGTTCACCGGGGTGGTGCCCATCCTGGTCGAGCTGGACGGCGACGTAAACGGCCACAAGTTCAGCGTGTCCGGCGAGGGCGAGGGCGATGCCACCTACGGCAAGCTGACCCTGAAGTTCATCTGCACCACCGGCAAGCTGCCCGTGCCCTGGCCCACCCTCGTGACCACCCTGACCTACGGCGTGCAGTGCTTCAGCCGCTACCCCGACCACATGAAGCAGCACGACTTCTTCAAGTCCGCCATGCCCGAAGGCTACGTCCAGGAGCGCACCATCTTCTTCAAGGACGACGGCAACTACAAGACCCGCGCCGAGGTGAAGTTCGAGGGCGACACCCTGGTGAACCGCATCGAGCTGAAGGGCATCGACTTCAAGGAGGACGGCAACATCCTGGGGCACAAGCTGGAGTACAACTACAACAGCCACAACGTCTATATCATGGCCGACAAGCAGAAGAACGGCATCAAGGTGAACTTCAAGATCCGCCACAACATCGAGGACGGCAGCGTGCAGCTCGCCGACCACTACCAGCAGAACACCCCCATCGGCGACGGCCCCGTGCTGCTGCCCGACAACCACTACCTGAGCACCCAGTCCGCCCTGAGCAAAGACCCCAACGAGAAGCGCGATCACATGGTCCTGCTGGAGTTCGTGACCGCCGCCGGGATCACTCTCGGCATGGACGAGCTGTACAAGTGACTCGAGCACCACCACCACCACCACTGAGATCCGGCTGCTAACAAAGCCCGAAAGGAAGCTGAGTTGGCTGCTGCCACCGCTGAGCAATAACTAGCATAACCCCTTGGGGCCTCTAAACGGGTCTTGAGGGGTTTTTTGCTGAAAGGAGGAACTATATCCGGAT |
| 3 | pET-28a-CaM::mCherry | TGGCGAATGGGACGCGCCCTGTAGCGGCGCATTAAGCGCGGCGGGTGTGGTGGTTACGCGCAGCGTGACCGCTACACTTGCCAGCGCCCTAGCGCCCGCTCCTTTCGCTTTCTTCCCTTCCTTTCTCGCCACGTTCGCCGGCTTTCCCCGTCAAGCTCTAAATCGGGGGCTCCCTTTAGGGTTCCGATTTAGTGCTTTACGGCACCTCGACCCCAAAAAACTTGATTAGGGTGATGGTTCACGTAGTGGGCCATCGCCCTGATAGACGGTTTTTCGCCCTTTGACGTTGGAGTCCACGTTCTTTAATAGTGGACTCTTGTTCCAAACTGGAACAACACTCAACCCTATCTCGGTCTATTCTTTTGATTTATAAGGGATTTTGCCGATTTCGGCCTATTGGTTAAAAAATGAGCTGATTTAACAAAAATTTAACGCGAATTTTAACAAAATATTAACGTTTACAATTTCAGGTGGCACTTTTCGGGGAAATGTGCGCGGAACCCCTATTTGTTTATTTTTCTAAATACATTCAAATATGTATCCGCTCATGAATTAATTCTTAGAAAAACTCATCGAGCATCAAATGAAACTGCAATTTATTCATATCAGGATTATCAATACCATATTTTTGAAAAAGCCGTTTCTGTAATGAAGGAGAAAACTCACCGAGGCAGTTCCATAGGATGGCAAGATCCTGGTATCGGTCTGCGATTCCGACTCGTCCAACATCAATACAACCTATTAATTTCCCCTCGTCAAAAATAAGGTTATCAAGTGAGAAATCACCATGAGTGACGACTGAATCCGGTGAGAATGGCAAAAGTTTATGCATTTCTTTCCAGACTTGTTCAACAGGCCAGCCATTACGCTCGTCATCAAAATCACTCGCATCAACCAAACCGTTATTCATTCGTGATTGCGCCTGAGCGAGACGAAATACGCGATCGCTGTTAAAAGGACAATTACAAACAGGAATCGAATGCAACCGGCGCAGGAACACTGCCAGCGCATCAACAATATTTTCACCTGAATCAGGATATTCTTCTAATACCTGGAATGCTGTTTTCCCGGGGATCGCAGTGGTGAGTAACCATGCATCATCAGGAGTACGGATAAAATGCTTGATGGTCGGAAGAGGCATAAATTCCGTCAGCCAGTTTAGTCTGACCATCTCATCTGTAACATCATTGGCAACGCTACCTTTGCCATGTTTCAGAAACAACTCTGGCGCATCGGGCTTCCCATACAATCGATAGATTGTCGCACCTGATTGCCCGACATTATCGCGAGCCCATTTATACCCATATAAATCAGCATCCATGTTGGAATTTAATCGCGGCCTAGAGCAAGACGTTTCCCGTTGAATATGGCTCATAACACCCCTTGTATTACTGTTTATGTAAGCAGACAGTTTTATTGTTCATGACCAAAATCCCTTAACGTGAGTTTTCGTTCCACTGAGCGTCAGACCCCGTAGAAAAGATCAAAGGATCTTCTTGAGATCCTTTTTTTCTGCGCGTAATCTGCTGCTTGCAAACAAAAAAACCACCGCTACCAGCGGTGGTTTGTTTGCCGGATCAAGAGCTACCAACTCTTTTTCCGAAGGTAACTGGCTTCAGCAGAGCGCAGATACCAAATACTGTCCTTCTAGTGTAGCCGTAGTTAGGCCACCACTTCAAGAACTCTGTAGCACCGCCTACATACCTCGCTCTGCTAATCCTGTTACCAGTGGCTGCTGCCAGTGGCGATAAGTCGTGTCTTACCGGGTTGGACTCAAGACGATAGTTACCGGATAAGGCGCAGCGGTCGGGCTGAACGGGGGGTTCGTGCACACAGCCCAGCTTGGAGCGAACGACCTACACCGAACTGAGATACCTACAGCGTGAGCTATGAGAAAGCGCCACGCTTCCCGAAGGGAGAAAGGCGGACAGGTATCCGGTAAGCGGCAGGGTCGGAACAGGAGAGCGCACGAGGGAGCTTCCAGGGGGAAACGCCTGGTATCTTTATAGTCCTGTCGGGTTTCGCCACCTCTGACTTGAGCGTCGATTTTTGTGATGCTCGTCAGGGGGGCGGAGCCTATGGAAAAACGCCAGCAACGCGGCCTTTTTACGGTTCCTGGCCTTTTGCTGGCCTTTTGCTCACATGTTCTTTCCTGCGTTATCCCCTGATTCTGTGGATAACCGTATTACCGCCTTTGAGTGAGCTGATACCGCTCGCCGCAGCCGAACGACCGAGCGCAGCGAGTCAGTGAGCGAGGAAGCGGAAGAGCGCCTGATGCGGTATTTTCTCCTTACGCATCTGTGCGGTATTTCACACCGCATATATGGTGCACTCTCAGTACAATCTGCTCTGATGCCGCATAGTTAAGCCAGTATACACTCCGCTATCGCTACGTGACTGGGTCATGGCTGCGCCCCGACACCCGCCAACACCCGCTGACGCGCCCTGACGGGCTTGTCTGCTCCCGGCATCCGCTTACAGACAAGCTGTGACCGTCTCCGGGAGCTGCATGTGTCAGAGGTTTTCACCGTCATCACCGAAACGCGCGAGGCAGCTGCGGTAAAGCTCATCAGCGTGGTCGTGAAGCGATTCACAGATGTCTGCCTGTTCATCCGCGTCCAGCTCGTTGAGTTTCTCCAGAAGCGTTAATGTCTGGCTTCTGATAAAGCGGGCCATGTTAAGGGCGGTTTTTTCCTGTTTGGTCACTGATGCCTCCGTGTAAGGGGGATTTCTGTTCATGGGGGTAATGATACCGATGAAACGAGAGAGGATGCTCACGATACGGGTTACTGATGATGAACATGCCCGGTTACTGGAACGTTGTGAGGGTAAACAACTGGCGGTATGGATGCGGCGGGACCAGAGAAAAATCACTCAGGGTCAATGCCAGCGCTTCGTTAATACAGATGTAGGTGTTCCACAGGGTAGCCAGCAGCATCCTGCGATGCAGATCCGGAACATAATGGTGCAGGGCGCTGACTTCCGCGTTTCCAGACTTTACGAAACACGGAAACCGAAGACCATTCATGTTGTTGCTCAGGTCGCAGACGTTTTGCAGCAGCAGTCGCTTCACGTTCGCTCGCGTATCGGTGATTCATTCTGCTAACCAGTAAGGCAACCCCGCCAGCCTAGCCGGGTCCTCAACGACAGGAGCACGATCATGCGCACCCGTGGGGCCGCCATGCCGGCGATAATGGCCTGCTTCTCGCCGAAACGTTTGGTGGCGGGACCAGTGACGAAGGCTTGAGCGAGGGCGTGCAAGATTCCGAATACCGCAAGCGACAGGCCGATCATCGTCGCGCTCCAGCGAAAGCGGTCCTCGCCGAAAATGACCCAGAGCGCTGCCGGCACCTGTCCTACGAGTTGCATGATAAAGAAGACAGTCATAAGTGCGGCGACGATAGTCATGCCCCGCGCCCACCGGAAGGAGCTGACTGGGTTGAAGGCTCTCAAGGGCATCGGTCGAGATCCCGGTGCCTAATGAGTGAGCTAACTTACATTAATTGCGTTGCGCTCACTGCCCGCTTTCCAGTCGGGAAACCTGTCGTGCCAGCTGCATTAATGAATCGGCCAACGCGCGGGGAGAGGCGGTTTGCGTATTGGGCGCCAGGGTGGTTTTTCTTTTCACCAGTGAGACGGGCAACAGCTGATTGCCCTTCACCGCCTGGCCCTGAGAGAGTTGCAGCAAGCGGTCCACGCTGGTTTGCCCCAGCAGGCGAAAATCCTGTTTGATGGTGGTTAACGGCGGGATATAACATGAGCTGTCTTCGGTATCGTCGTATCCCACTACCGAGATATCCGCACCAACGCGCAGCCCGGACTCGGTAATGGCGCGCATTGCGCCCAGCGCCATCTGATCGTTGGCAACCAGCATCGCAGTGGGAACGATGCCCTCATTCAGCATTTGCATGGTTTGTTGAAAACCGGACATGGCACTCCAGTCGCCTTCCCGTTCCGCTATCGGCTGAATTTGATTGCGAGTGAGATATTTATGCCAGCCAGCCAGACGCAGACGCGCCGAGACAGAACTTAATGGGCCCGCTAACAGCGCGATTTGCTGGTGACCCAATGCGACCAGATGCTCCACGCCCAGTCGCGTACCGTCTTCATGGGAGAAAATAATACTGTTGATGGGTGTCTGGTCAGAGACATCAAGAAATAACGCCGGAACATTAGTGCAGGCAGCTTCCACAGCAATGGCATCCTGGTCATCCAGCGGATAGTTAATGATCAGCCCACTGACGCGTTGCGCGAGAAGATTGTGCACCGCCGCTTTACAGGCTTCGACGCCGCTTCGTTCTACCATCGACACCACCACGCTGGCACCCAGTTGATCGGCGCGAGATTTAATCGCCGCGACAATTTGCGACGGCGCGTGCAGGGCCAGACTGGAGGTGGCAACGCCAATCAGCAACGACTGTTTGCCCGCCAGTTGTTGTGCCACGCGGTTGGGAATGTAATTCAGCTCCGCCATCGCCGCTTCCACTTTTTCCCGCGTTTTCGCAGAAACGTGGCTGGCCTGGTTCACCACGCGGGAAACGGTCTGATAAGAGACACCGGCATACTCTGCGACATCGTATAACGTTACTGGTTTCACATTCACCACCCTGAATTGACTCTCTTCCGGGCGCTATCATGCCATACCGCGAAAGGTTTTGCGCCATTCGATGGTGTCCGGGATCTCGACGCTCTCCCTTATGCGACTCCTGCATTAGGAAGCAGCCCAGTAGTAGGTTGAGGCCGTTGAGCACCGCCGCCGCAAGGAATGGTGCATGCAAGGAGATGGCGCCCAACAGTCCCCCGGCCACGGGGCCTGCCACCATACCCACGCCGAAACAAGCGCTCATGAGCCCGAAGTGGCGAGCCCGATCTTCCCCATCGGTGATGTCGGCGATATAGGCGCCAGCAACCGCACCTGTGGCGCCGGTGATGCCGGCCACGATGCGTCCGGCGTAGAGGATCGAGATCTCGATCCCGCGAAATTAATACGACTCACTATAGGGGAATTGTGAGCGGATAACAATTCCCCTCTAGAAATAATTTTGTTTAACTTTAAGAAGGAGATATACCATGGGCAGCAGCCATCATCATCATCATCACAGCAGCGGCCTGGTGCCGCGCGGCAGCCATATGGCTAGCATGACTGGTGGACAGCAAATGGGTCGCGGATCCatggctGACCAACTGACTGAAGAGCAGATCGCAGAATTTAAAGAGGAATTCTCCCTATTTGACAAGGACGGGGATGGGACAATAACAACCAAGGAGCTGGGGACGGTGATGCGGTCTCTGGGGCAGAACCCCACAGAAGCAGAGCTGCAGGACATGATCAATGAAGTAGATGCCGACGGTGACGGCACAATCGACTTCCCTGAGTTCCTGACAATGATGGCAAGAAAAATGAAATACAGGGACACGGAAGAAGAAATTAGAGAAGCGTTCGGTGTGTTTGATAAGGATGGCAATGGCTACATCAGTGCAGCAGAGCTTCGCCACGTGATGACAAACCTTGGAGAGAAGTTAACAGATGAAGAGGTTGATGAAATGATCAGGGAAGCAGACATCGATGGGGATGGTCAGGTAAACTACGAAGAGTTTGTACAAATGATGACAGCGAAGttaCCGGTCGCCACCATGGTGAGCAAGGGCGAGGAGGATAACATGGCCATCATCAAGGAGTTCATGCGCTTCAAGGTGCACATGGAGGGCTCCGTGAACGGCCACGAGTTCGAGATCGAGGGCGAGGGCGAGGGCCGCCCCTACGAGGGCACCCAGACCGCCAAGCTGAAGGTGACCAAGGGTGGCCCCCTGCCCTTCGCCTGGGACATCCTGTCCCCTCAGTTCATGTACGGCTCCAAGGCCTACGTGAAGCACCCCGCCGACATCCCCGACTACTTGAAGCTGTCCTTCCCCGAGGGCTTCAAGTGGGAGCGCGTGATGAACTTCGAGGACGGCGGCGTGGTGACCGTGACCCAGGACTCCTCCCTGCAGGACGGCGAGTTCATCTACAAGGTGAAGCTGCGCGGCACCAACTTCCCCTCCGACGGCCCCGTAATGCAGAAGAAGACCATGGGCTGGGAGGCCTCCTCCGAGCGGATGTACCCCGAGGACGGCGCCCTGAAGGGCGAGATCAAGCAGAGGCTGAAGCTGAAGGACGGCGGCCACTACGACGCTGAGGTCAAGACCACCTACAAGGCCAAGAAGCCCGTGCAGCTGCCCGGCGCCTACAACGTCAACATCAAGTTGGACATCACCTCCCACAACGAGGACTACACCATCGTGGAACAGTACGAACGCGCCGAGGGCCGCCACTCCACCGGCGGCATGGACGAGCTGTACAAGTAGCGGCCGCACTCGAGCACCACCACCACCACCACTGAGATCCGGCTGCTAACAAAGCCCGAAAGGAAGCTGAGTTGGCTGCTGCCACCGCTGAGCAATAACTAGCATAACCCCTTGGGGCCTCTAAACGGGTCTTGAGGGGTTTTTTGCTGAAAGGAGGAACTATATCCGGAT |

**Supplementary Table 9.** The table shows the sequences of bacterial expression fusion constructs pET-28a-SH3-I_2_-GUK::GFP, pET-28a-SH3-I_3_-GUK::GFP and pET-28a-CaM::mCherry.
