# Supplementary material for: Nanoscale regulation of Ca2+ dependent phase transitions and real-time dynamics of SAP97/hDLG
Source: Nat Commun. 2022 Jul 22;13:4236. doi: 10.1038/s41467-022-31912-1 (PMC9307800; doi:10.1038/s41467-022-31912-1)
Supplement: Supplementary file 3 — Reporting Summary [file 41467_2022_31912_MOESM3_ESM.pdf]

nature portfolio

Corresponding author(s): Deepak Nair

Last updated by author(s): Jun 30, 2022

Reporting Summary

Nature Portfolio wishes to improve the reproducibility of the work that we publish. This form provides structure for consistency and transparency in reporting. For further information on Nature Portfolio policies, see our Editorial Policies and the Editorial Policy Checklist.

Statistics

For all statistical analyses, confirm that the following items are present in the figure legend, table legend, main text, or Methods section.

n/a	Confirmed	
☐	☒	The exact sample size (*n*) for each experimental group/condition, given as a discrete number and unit of measurement
☐	☒	A statement on whether measurements were taken from distinct samples or whether the same sample was measured repeatedly
☐	☒	The statistical test(s) used AND whether they are one- or two-sided<br>*Only common tests should be described solely by name; describe more complex techniques in the Methods section.*
☐	☒	A description of all covariates tested
☐	☒	A description of any assumptions or corrections, such as tests of normality and adjustment for multiple comparisons
☐	☒	A full description of the statistical parameters including central tendency (e.g. means) or other basic estimates (e.g. regression coefficient) AND variation (e.g. standard deviation) or associated estimates of uncertainty (e.g. confidence intervals)
☐	☒	For null hypothesis testing, the test statistic (e.g. *F*, *t*, *r*) with confidence intervals, effect sizes, degrees of freedom and *P* value noted<br>*Give P values as exact values whenever suitable.*
☒	☐	For Bayesian analysis, information on the choice of priors and Markov chain Monte Carlo settings
☒	☐	For hierarchical and complex designs, identification of the appropriate level for tests and full reporting of outcomes
☒	☐	Estimates of effect sizes (e.g. Cohen's *d*, Pearson's *r*), indicating how they were calculated

Our web collection on statistics for biologists contains articles on many of the points above.

Software and code

Policy information about availability of computer code

Data collection	SMLM and FRAP : MetaMorph (Molecular devices, San Jose, USA) software (Version 7.8.11.0) was used to steer the microscope and for data acquisition. Super resolution imaging was performed on the whole cell and FRAP was performed 10-25 micrometers from cell periphery.<br>QPCR : The data was acquired using Quant Studio 7Flex Real-time Q-PCR system software (Applied Biosystems, USA).<br>Confocal Microscopy : The equipment for data acquisition was controlled using ZEN 2011 LSM software (Carl Zeiss, Thornwood, USA).
Data analysis	FRAP : Y = Y0 + (Plateau−Y0) x (1−exp(−Kx)): Two tailed Welch's t-test for two sample comparison and Brown-Forsythe and Welch's ANOVA with Dunn's correction for multiple comparison. The analysis of the acquired data was performed in MetaMorph software (7.10.1.181) and GraphPad PRISM (8.4.2).<br>SMLM : The algorithm for quantitative data analysis of protein localization was performed using PALM-TRACER (PALMTRACERX64, 2017) plugin in MetaMorph software.<br>QPCR : The data was analyzed and compiled in Quant Studio 7Flex Real-time Q-PCR software.<br>Confocal Microscopy : The image analysis was performed in MetaMorph software (7.10.1.181).<br>d' Augustino-Pearson's Omnibus test for normality of the data<br>Free energy model : y=an^(2/3)+/-bn+c<br>Rank order analysis : y=mx+c<br>Cumulative distribution functions : K-S Test<br>The Rank-Order analysis of SAP97/hDLG clusters was performed using a code written in Jupyter notebook 5.6.0, to derive the scaling of average intensity and area of clusters. Public GitHub repository for the code can be found at [https://github.com/LucisQ/Rank-order.git].

For manuscripts utilizing custom algorithms or software that are central to the research but not yet described in published literature, software must be made available to editors and reviewers. We strongly encourage code deposition in a community repository (e.g. GitHub). See the Nature Portfolio guidelines for submitting code & software for further information.

Data

Policy information about availability of data

All manuscripts must include a data availability statement. This statement should provide the following information, where applicable:

- Accession codes, unique identifiers, or web links for publicly available datasets
- A description of any restrictions on data availability
- For clinical datasets or third party data, please ensure that the statement adheres to our policy

All data associated with this study are in the paper and/or the Supplementary Information. All the mathematical and statistical tools that are used for analysis is available in the public domain. Source data are provided as a Source Data file with this manuscript.

Field-specific reporting

Please select the one below that is the best fit for your research. If you are not sure, read the appropriate sections before making your selection.

☒ Life sciences ☐ Behavioural & social sciences ☐ Ecological, evolutionary & environmental sciences

For a reference copy of the document with all sections, see nature.com/documents/nr-reporting-summary-flat.pdf

Life sciences study design

All studies must disclose on these points even when the disclosure is negative.

Sample size	All the sample sizes were calculated using G*Power (Version 3.1.9.7). For the morphometric data from SMLM, sample sizes were calculated using effect size of 0.1, α error prob of 0.1, power of 0.9 from 2-4 groups. For the FRAP experiments, sample sizes were calculated using effect size of 0.7, α error prob of 0.1, power of 0.9 for 2-4 groups.<br>SMLM : N= 800-1200 clusters from 10 cells (Biological Replicates 3) per condition. (Venkatesan et al., 2020)<br>FRAP : N = 13 cells; 26 ROI (Control), 8 cells; 13 ROI (Tg), 8 cells; 12 ROI (w7) and 9 cells; 18 ROI, 3 Biological Replicates. (Faul et al, 2007, Faul et al 2009)<br>QPCR : N= 9 cortex, 18 Hippocampus (9 independent animals) (Faul et al, 2007, Faul et al 2009)<br>3 passages of Neuro-2a cells (ATCC, CCL131)
Data exclusions	SMLM : For Rank Order analysis, 80% of the data points were included in the analysis to reduce the computational load. Multiple fractions of data was selected and tested for significance and the data above 80% showed no significant difference.
Replication	All measurements are collected from at least three Biological replicates. A minimum of 2 technical replicates were chosen for the experiments from every biological replicates
Randomization	The samples were randomly chosen for all the experimental groups.
Blinding	Semi automated analysis of samples were performed for single molecule detection, rank order analysis and extraction of nanoscale clusters. Analysis is partially blinded where only single molecule thresholds for segmentation are provided by user and this threshold kept constant between different samples. Rest of the analysis every step remains constant for all the data as described in materials and methods with no additional manual intervention or sorting of the data.

Behavioural & social sciences study design

All studies must disclose on these points even when the disclosure is negative.

Study description	*Briefly describe the study type including whether data are quantitative, qualitative, or mixed-methods (e.g. qualitative cross-sectional, quantitative experimental, mixed-methods case study).*
Research sample	*State the research sample (e.g. Harvard university undergraduates, villagers in rural India) and provide relevant demographic information (e.g. age, sex) and indicate whether the sample is representative. Provide a rationale for the study sample chosen. For studies involving existing datasets, please describe the dataset and source.*
Sampling strategy	*Describe the sampling procedure (e.g. random, snowball, stratified, convenience). Describe the statistical methods that were used to predetermine sample size OR if no sample-size calculation was performed, describe how sample sizes were chosen and provide a rationale for why these sample sizes are sufficient. For qualitative data, please indicate whether data saturation was considered, and what criteria were used to decide that no further sampling was needed.*
Data collection	*Provide details about the data collection procedure, including the instruments or devices used to record the data (e.g. pen and paper, computer, eye tracker, video or audio equipment) whether anyone was present besides the participant(s) and the researcher, and whether the researcher was blind to experimental condition and/or the study hypothesis during data collection.*
Timing	*Indicate the start and stop dates of data collection. If there is a gap between collection periods, state the dates for each sample*

Timing	*cohort.*
Data exclusions	*If no data were excluded from the analyses, state so OR if data were excluded, provide the exact number of exclusions and the rationale behind them, indicating whether exclusion criteria were pre-established.*
Non-participation	*State how many participants dropped out/declined participation and the reason(s) given OR provide response rate OR state that no participants dropped out/declined participation.*
Randomization	*If participants were not allocated into experimental groups, state so OR describe how participants were allocated to groups, and if allocation was not random, describe how covariates were controlled.*

Ecological, evolutionary & environmental sciences study design

All studies must disclose on these points even when the disclosure is negative.

Study description	*Briefly describe the study. For quantitative data include treatment factors and interactions, design structure (e.g. factorial, nested, hierarchical), nature and number of experimental units and replicates.*
Research sample	*Describe the research sample (e.g. a group of tagged Passer domesticus, all Stenocereus thurberi within Organ Pipe Cactus National Monument), and provide a rationale for the sample choice. When relevant, describe the organism taxa, source, sex, age range and any manipulations. State what population the sample is meant to represent when applicable. For studies involving existing datasets, describe the data and its source.*
Sampling strategy	*Note the sampling procedure. Describe the statistical methods that were used to predetermine sample size OR if no sample-size calculation was performed, describe how sample sizes were chosen and provide a rationale for why these sample sizes are sufficient.*
Data collection	*Describe the data collection procedure, including who recorded the data and how.*
Timing and spatial scale	*Indicate the start and stop dates of data collection, noting the frequency and periodicity of sampling and providing a rationale for these choices. If there is a gap between collection periods, state the dates for each sample cohort. Specify the spatial scale from which the data are taken*
Data exclusions	*If no data were excluded from the analyses, state so OR if data were excluded, describe the exclusions and the rationale behind them, indicating whether exclusion criteria were pre-established.*
Reproducibility	*Describe the measures taken to verify the reproducibility of experimental findings. For each experiment, note whether any attempts to repeat the experiment failed OR state that all attempts to repeat the experiment were successful.*
Randomization	*Describe how samples/organisms/participants were allocated into groups. If allocation was not random, describe how covariates were controlled. If this is not relevant to your study, explain why.*
Blinding	*Describe the extent of blinding used during data acquisition and analysis. If blinding was not possible, describe why OR explain why blinding was not relevant to your study.*

Did the study involve field work? ☐ Yes ☐ No

Field work, collection and transport

Field conditions	*Describe the study conditions for field work, providing relevant parameters (e.g. temperature, rainfall).*
Location	*State the location of the sampling or experiment, providing relevant parameters (e.g. latitude and longitude, elevation, water depth).*
Access & import/export	*Describe the efforts you have made to access habitats and to collect and import/export your samples in a responsible manner and in compliance with local, national and international laws, noting any permits that were obtained (give the name of the issuing authority, the date of issue, and any identifying information).*
Disturbance	*Describe any disturbance caused by the study and how it was minimized.*

Reporting for specific materials, systems and methods

We require information from authors about some types of materials, experimental systems and methods used in many studies. Here, indicate whether each material, system or method listed is relevant to your study. If you are not sure if a list item applies to your research, read the appropriate section before selecting a response.

Materials & experimental systems

n/a	Involved in the study
☐	☒ Antibodies
☐	☒ Eukaryotic cell lines
☒	☐ Palaeontology and archaeology
☐	☒ Animals and other organisms
☒	☐ Human research participants
☒	☐ Clinical data
☒	☐ Dual use research of concern

Methods

n/a	Involved in the study
☒	☐ ChIP-seq
☒	☐ Flow cytometry
☒	☐ MRI-based neuroimaging

Antibodies

Antibodies used

SAP97 antibody- APZ-010, Alomone Labs, Jerusalem (1:500 dilution in 3% BSA).
PSD95 antibody MA1 046, Invitrogen, USA (1:500 dilution in 3% BSA).
Alexa Fluor 647 (anti-Rabbit) and Alexa fluor 532 (anti-Mouse), Invitrogen, USA (1:500 dilution in 3% BSA)

Validation

The SAP97 antibody was validated by immuno-blotting and the PSD95 antibody was validated previously by Nair et al.,2013

Eukaryotic cell lines

Policy information about cell lines

Cell line source(s)

Neuro-2a, ATCC® CCL-131™

Authentication

Commercially available Cell line, Not authentificated

Mycoplasma contamination

Mycoplama tests routinely done to rule out contamination

Commonly misidentified lines
(See ICLAC register)

No commonly misidentified cell lines were used in the study

Animals and other organisms

Policy information about studies involving animals; ARRIVE guidelines recommended for reporting animal research

Laboratory animals

Mus musculus, mixed sex, BL-6, Postnatal-0 day old pups (N=9 pups). Rattus norwegicus, mixed sex Srague-Dawley, Postnatal-0 day old pups (N=9 pups).

Wild animals

No wild animals were used in the study.

Field-collected samples

No field collected samples were used in the study.

Ethics oversight

Central Animal Facility, Indian Institute of Science, Institute Bio safety and Ethics approval Committee
All the procedures in this study were performed according to the rules and guidelines declared in the Compendium of CPCSEA 2018 by the Committee for the Purpose of Control and Supervision of Experimental Animals (CPCSEA), Ministry of Fisheries, Animal Husbandry and Dairying, India. The research protocol (CAF/Ethics/659 and CAF/Ethics/790) was approved by the Institutional Animal Ethics Committee (IAEC) of the Indian Institute of Science.

Note that full information on the approval of the study protocol must also be provided in the manuscript.
